# Cohen’s Conservatism and Human Enhancement

**DOI:** 10.1007/s10892-013-9151-0

**Published:** 2013-10-24

**Authors:** Jonathan Pugh, Guy Kahane, Julian Savulescu

**Affiliations:** Oxford Uehiro Centre for Practical Ethics, Suite 8, Littlegate House, St Ebbes Street, Oxford, OX1 1PT UK

**Keywords:** G. A. Cohen, Conservatism, Enhancement, Value

## Abstract

In an intriguing essay, G. A. Cohen has defended a conservative bias in favour of existing value. In this paper, we consider whether Cohen’s conservatism raises a new challenge to the use of human enhancement technologies. We develop some of Cohen’s suggestive remarks into a new line of argument against human enhancement that, we believe, is in several ways superior to existing objections. However, we shall argue that on closer inspection, Cohen’s conservatism fails to offer grounds for a strong sweeping objection to enhancement, and may even offer *positive* support for forms of enhancement that preserve valuable features of human beings. Nevertheless, we concede that Cohen’s arguments may suggest some plausible and important constraints on the *modality* of legitimate and desirable enhancements.

It has become apparent that we may soon be able to use pharmacological and genetic technologies to enhance human traits, when that is understood to refer to the improvement of human form or functioning “… beyond what is necessary to sustain or restore good health” (Juengst 1998, p. 29). The use of these technologies for the purposes of human enhancement has met with a wide variety of moral objections (The President’s Council on Bioethics 2003; Elliott 2003; Kass 2003; Sandel 2007). In this paper, we investigate a potentially powerful new objection to human enhancement suggested by the late G. A. Cohen shortly before his death in his defence of the conservative attitude of having a “… bias in favour of retaining what is of value, even in the face of replacing it by something of greater value” (Cohen 2011, p. 203). Following Cohen, we shall henceforth refer to this attitude as ‘the conservative bias’.

We shall consider five different types of human enhancement as examples to which Cohen’s objection might apply. First, we might seek to *cognitively* enhance agents with even an above average IQ by providing them with certain drugs (such as Modafinil) that increase cognitive abilities. Second, we might seek to *morally* enhance agents by providing them with drugs that enhance moral attitudes such as altruism and empathy. Third, selective serotonin reuptake inhibitors offer the possibility of *mood* enhancement insofar as they can improve even a healthy person’s general mood and ability to interact socially. Fourthly, we might soon be able to use new technologies to extend a person’s *lifespan* well beyond the current norm. Finally, we may be able to modify the 3 phases of human love—lust, attraction and attachment—*enhancing love* itself (Savulescu and Sandberg 2008) and even reducing loving feelings in abusive relationships (Earp et al. 2012).

The thrust of Cohen’s argument in defence of the conservative bias is that we ought to preserve extant valuable things, even in favour of possible replacements which would possess more of the same value. As we shall show, Cohen himself recognised and highlighted the possibility of an objection to human enhancement based on his general defence of the conservative bias. The conservative nature of the objection that can be drawn from Cohen’s thought here is of particular interest when we acknowledge the otherwise progressive nature of his canon of work (Cohen 1978, 2009); it shows that opposition to human enhancement technologies is not merely the preserve of right wing bio-conservatives who have so far spearheaded the opposition to the use of these technologies.

We shall show that Cohen’s striking claims about value do suggest an interesting new line of argument against enhancement that is in several ways clearer and superior to existing objections. However, we shall argue that on closer inspection, this argument fails to offer valid grounds for a strong sweeping objection to enhancement. We shall further suggest that Cohen’s defence of conservatism with respect to existing value may actually amount to the recognition of the role of *history* in generating current value. In view of this, we shall claim that although Cohen’s arguments suggest some plausible and important new constraints on the *modality* of legitimate and desirable enhancements, they do not rule out the moral permissibility of *all* human enhancements, and may even offer positive support for some forms of enhancement. Before we proceed, let us note that although we shall focus on the question of enhancement, much of what we say should also apply to attempts to apply Cohen’s conservatism in other domains.

We shall begin the paper by briefly delineating Cohen’s thesis concerning conservatism with respect to value, before going on to explain how this thesis might pertain to the use of enhancement technologies. We shall then consider how an objection to the use of enhancement technologies may be developed from Cohen’s thesis.

## Conserving the Valuable, Conserving the Valued and the Conservative Bias

In defending the conservative bias, Cohen draws a distinction between two types of valuing that the conservative endorses, namely *valuing the valuable*, and *valuing the valued*. We shall first delineate Cohen’s understanding of these two types of valuing before explaining how he uses them in his defence of the conservative bias identified above.

It is perhaps instructive to begin by first considering the overall approach to valuing that the conservative (in Cohen’s sense) opposes. The approach to valuing that the conservative opposes according to Cohen is that which adopts an attitude towards *bearers* of value whereby they.

… do not count as such, but matter only because of the value that they bear, and are therefore, in a deep sense dispensible. (Cohen 2011, p. 212).

Such an approach is most familiarly a salient feature of maximizing, monistic value theories such as classical utilitarianism, although it is also often assumed by non-maximizing and pluralistic value theories (Cohen 2011, p. 212). The central thought underlying this approach is that the only thing that matters with regards to extant bearers of value is the value that they bear; their being a particular existing entity is of no consequence with respect to their value. As such, if it is possible to bring about more of the value that the extant entity bears by replacing it with something else, then on this approach to valuing, we should do so.^1^ In view of some of the other terminology that Cohen goes on to use, we will call this approach *valuing value.*

In contrast to this approach, Cohen claims, in a conservative vein, that we ought to value extant bearers of value *over and above* the value that they bear, on the basis that they already exist and bear value. Cohen identifies two ways in which we can adopt this approach to valuing. The first he terms *valuing the valuable*. In this type of valuing, the object of one’s evaluation is a particular existing entity which is intrinsically valuable. The value of such an object is not contingent upon the evaluator’s attitudes, or their specific relation to that object; rather, the particular object is valuable, *qua* particular, in and of itself (Cohen 2011, pp. 206–207 and 210–211). For example, we might say that a beautiful piece of art such as Michelangelo’s sculpture of David instantiates this sort of value.

In addition, and in contrast to valuing the valuable, Cohen also discusses what he terms *valuing the valued*. Here, the object of the agent’s evaluation need not have any intrinsic value; rather the main source of the object’s value lies in its particular relation to the evaluator. For example, it might be claimed that the reason that brides often retain their wedding dresses after their wedding day is that they instantiate this sort of personal value. On Cohen’s interpretation, even if the dress can be said to bear a degree of intrinsic value, the main reason that the bride will value the dress is because of her specific relationship to *that* dress.^2^

In contrast to what we have termed ‘valuing value’, Cohen claims that the conservative approaches of ‘valuing the valued’ and ‘valuing the valuable’ share in the fact that they involve valuing something “other than solely on account of the amount and type of value that resides in that thing” (Cohen 2011, p. 207). However, there is an important difference between ‘valuing the valuable’ and ‘valuing the valued’, which allows Cohen to appeal to these different modes of valuing in order to defend the conservative bias in two different ways. As such, we shall now highlight the difference between these two types of conservative modes of valuing before explaining how Cohen goes on to defend the conservative bias by appealing to each activity.

The difference between these two types of valuing lies in the fact that the claim that ‘both types of valuing involve valuing something other than solely on account of the value that resides in that thing’ is true with regards to each type of valuing for different reasons. On the one hand, the preceding claim is true of ‘valuing the valuable’ because even though the object of this type of valuing has intrinsic value, our valuing that object is not (according to Cohen) merely our valuing the intrinsic value which that object instantiates; rather, we also value the particular object itself that instantiates the value *qua* particular (Cohen 2011, p. 207). To illustrate, on Cohen’s view, although we may say that Michelangelo’s David bears a certain intrinsic value, we should also value the particular extant physical entity that bears that intrinsic value. In view of this, he comes to term the value involved in ‘valuing the valuable’ which is not merely the intrinsic value that the object bears, as ‘particular value’.

The claim that ‘valuing the valued’ involves valuing something other than solely on account of the amount and type of value that resides in that thing is perhaps more obviously true. After all, the object of such valuing may have either very little intrinsic value or even no intrinsic value at all; as we saw in the example of the wedding dress, the value of the object lies not in the object itself, at least not mainly; rather the dress’s value lies in its specific relation to the evaluator. In view of this, Cohen comes to term the sort of value involved in ‘valuing the valued’ as ‘personal value’. We might also highlight here that, although Cohen himself does not point this out, it seems plausible to claim that the relationship of the evaluator to the valued item is based in part upon a shared personal history with that item.^3^

There may be other ways to understand the special relation of the bride to her wedding dress. For example, rather than appealing to the concept of personal value, rights-based accounts might explain the wedding dress example by focusing on the rights that agents have to what belongs to them (grounded perhaps in the value of self-ownership), and on our reasons to respect the desires of other autonomous agents.^4^ For example, it might be claimed that the moral reason that we have to honour a bride’s keeping her wedding dress is not that the dress has some special ‘conservative’ sort of personal value, but simply because it is *the bride’s dress*, and it is therefore something that *she* has the right to.^5^

The rights based approach is an interesting alternative way of explaining aspects of this case. However, it is not clear that this alternative is preferable to Cohen’s interpretation. Indeed, Cohen himself provided objections to the self-ownership principle that underlies prominent rights-based accounts of the sort just outlined.^6^ As such, whilst the rights based approach will serve as a useful comparison for some of the cases that we shall come to consider, our focus in this paper is on the distinctive claims made by Cohen’s conservative axiology. However, the contrast with a rights-based approach highlights one interesting feature of Cohen’s view: it purports to give us reasons to refrain from acting in ways that would maximise value, not by appealing to rights or other deontological constraints, but by asking us to rethink our understanding of value itself.

To return to Cohen’s account, with the distinction between particular and personal value in mind, we can understand how Cohen defends the conservative bias by appealing to these two types of value. Consider first the case of particular value. Cohen argues that if we hold the attitude that we should always replace an existing object which bears less intrinsic value with an object which bears more of the same intrinsic value, we in fact *devalue* the particular existent things that bear intrinsic value; that is to say, we fail to recognise the particular value of extant value bearers.

To illustrate, consider the following example. Suppose that Michelangelo’s David had been made of an extremely rare material which afforded the artist a completely unique opportunity for artistic expression through sculpture. Suppose, quite fantastically, that Michelangelo had completed David but later realised that he could have made an even more beautiful sculpture, say of Eve, if he had used that material. And assume that at some later point, Michelangelo was actually in a position to destroy David and use the material to create the even better Eve. Moreover, we could be absolutely certain that he was right; Eve would be better. Finally, we may assume that he would not fail to produce the new sculpture, and that everybody agreed that the promised sculpture would bear more of the same intrinsic value that Michelangelo’s David bears. The question then is should we destroy Michelangelo’s David in order to bring about a sculpture which bears more intrinsic value?

According to Cohen’s thesis, the answer should be no. Agreeing to destroy Michelangelo’s David in order to bring about more intrinsic value in Eve is to fail to recognise the particular value of Michelangelo’s existing sculpture of David. We should, according to Cohen, hold a bias towards extant particulars which bear intrinsic value, even in the face of replacement particulars which would bear more of the same intrinsic value.^7^ Accordingly, on Cohen’s view, rather than prioritising the maximization value per se (as per the non-conservative approach of ‘valuing value’), we ought to have a bias in favour of conserving the things that already exist and bear value.

To take a more mundane example, a family may have an old dog, Jessie, who they value as a member of the family, but who is now slow and labouring due to arthritis. They are offered a new puppy by a friend, Sally. The new dog would be friendlier, cuter and more active, being able to play with the younger children more. They could put Jessie down and accept Sally. However, even if Sally would make a better family pet, accepting her would be to fail to value the valued, and perhaps even to fail to value the valuable. To extend the point, a similar thought could be said to apply to the value of present over future generations, or to the choice between extending the life of existing persons versus replacing them with a new generation. In both these cases, there are reasons based on particular and personal value to give priority to extant holders of value.

Having stated this, Cohen does acknowledge that the conservative bias is defeasible.^8^ If the value of the new objects which replace the old is of a sufficient magnitude, Cohen suggests that it may still be correct to bring them about. For example, the dog, Jessie, might be blind, deaf and very ill now, perhaps in pain. However, the important caveat in such a case is that we still ought to lament the existent value that we have lost in doing so. In this latter case, Cohen’s view does not alter the moral outcome, but rather our moral understanding of that outcome; although we may rejoice the greater value that has been created, this attitude should also involve a lamentation of the value we have also lost in doing so.

The conservative bias can also be defended by appealing to the nature of personal value. Again, the way in which personal value can be used to defend the conservative bias is perhaps more obvious. It is a common feature of our experience to become attached to certain objects which might have no real intrinsic value, but which we would still claim to be irreplaceable; we often claim that such objects have sentimental value. Consider again the woman who keeps her wedding dress after her wedding. Even if she could replace it with a far more expensive and beautiful dress, this would not be a good reason to do so; the replacement would lack the relation of the old wedding dress to the woman and her personal history with it.^9^ Accordingly, it seems that we have a reason to hold a conservative bias in favour of those things which instantiate personal value over those which instantiate greater value in other ways but lack personal value.^10^

One difference between personal and particular value that Cohen does not stress is that the relationships between agents and certain extant objects which undergird the concept of personal value often seems to be grounded in the shared personal history with the extant valued object that ties us to that thing.^11^ When we place personal value on something, it seems more accurate to say that it is our shared heritage with the object that matters, and not the mere fact that the object exists.^12^

Moreover, it seems that our intuitions about (or reasons associated with) personal value involving shared history are far stronger than those provided by particular value. To bring this thought out, recall the hypothetical example of Michelangelo creating a sculpture of Eve. This time, suppose that his creation of the even better Eve would not require the destruction of David; there is just enough material left for one more beautiful sculpture, which Michelangelo then uses to create Eve. However, immediately afterwards, Michelangelo announces that he has a better idea for creating an even better sculpture than Eve. But its creation would require the destruction of the newly-created sculpture, Eve.

Here, there intuitively seems to be a much better case for replacing the recently created sculpture than there was in the case of David above. Part of the reason seems to be that the Eve lacks the unique heritage of Michelangelo’s David as we know him, which has long been in view and the object of admiration. In the case of Eve and her superior successor, both sculptures would have the same history, apart from the fact that the successor would be made shortly afterwards, and involve destroying the Eve. The reason that we believe that Michelangelo’s David ought to be preserved in similar circumstances is that we place *personal* value on the existing sculpture by virtue of its unique heritage, and its unique relation to human artistic endeavour. The fact that we believe it to be irreplaceable does not seem to be an upshot of the intrinsic value it instantiates, nor the fact that it merely exists and instantiates *particular* value; rather, we believe that the existing sculpture is irreplaceable because we personally value it by virtue of its unique history. Accordingly, it seems that the reasons to conserve provided by personal value are typically stronger than reasons provided by particular value, due in part to the role that history plays in tying us to the valued entity.

These striking claims about value have great intuitive force. However, as Cohen himself acknowledged, they also face some difficulties (Cohen 2011, pp. 214–221).^13^ In what follows, we will not press such worries. Instead, we want to explore whether Cohen’s claims about particular and personal value can be used to mount a forceful new objection to the project of enhancement. If such an objection can be mounted, it would be superior in important ways to more familiar bio-conservative worries, which have attracted significant response (Harris 2007; Buchanan’s 2011; Kamm 2005; Savulescu 2009). These more familiar bioconservative worries either explicitly draw on religion, or rely on notions that often have their source in religion. Cohen’s claims about value do not have this problematic baggage. Moreover, these claims are not based in an attempt to spell out (or rationalize) one’s repugnance or gut rejection of enhancement. They are rather a general account of value that was motivated independently and is only later applied to the enhancement case. Moreover, as we shall see, Cohen’s conservatism may suggest reasons to preserve our human nature that do not require appeal to the implausible and obscure idea that some things are wrong simply because they are ‘unnatural’.

## The Conservative Bias and Human Enhancement

On the face of it, Cohen’s views seem to supply powerful ammunition to opponents of enhancement. They would supply such ammunition even if Cohen never discussed the question of enhancement. However, Cohen himself clearly thought of enhancement as a paradigmatic example of the kind of threat to value that he highlights. But Cohen’s remarks on this are rather brief and sketchy. In this section, we shall try to flesh out the objection that can be drawn from Cohen’s remarks.

The first suggestive comment that Cohen makes with regards to enhancement technologies is his claim that most people’s intuitive response to one example of what would qualify as an enhancement shows that, in practice, “… everybody is conservative to some degree” (Cohen 2011, p.208). He sets out the example as follows:

… there sits a man who is surveying his own fleshy parts, that is, those of his parts which are still made of flesh, which includes some of his brain-flesh parts, and he is replacing defective bits of his flesh by perfect artificial substitutes… The man has been doing this for some time, and a lot of him is already artificial. That is surely a ghastly scenario. (Cohen 2011, p. 208)

Whilst it is open to question whether this is really a ‘ghastly’ scenario, one reason that explains why some might find it to be so (according to Cohen) is that we accept the broad conservative claim that… “certain things are to be taken as they come; they are not to be shaped or controlled” (Cohen 2011, p. 208). This claim is similar to that which underlies a prominent objection to human enhancement technologies, voiced most famously by Michael Sandel, namely the objection that we ought to accept some things as ‘given’ and that the main problem with enhancement is a Promethean drive to mastery (Sandel 2007). Cohen in fact also distinguishes the activity of ‘valuing the given’ from the two types of conservative value presented here (Cohen 2011, p. 207). Although Cohen appears to endorse this additional mode of valuing, we shall not consider it directly here because, as we have already mentioned, it is already associated with a prominent objection to human enhancement which has been discussed extensively (Kamm 2005). However, our consideration of the other types of valuing that Cohen identifies will also have some application to such appeals to the value of ‘the given’.

Cohen goes on to consider a further example of enhancement. He writes:

If I want us to continue as we are, do I want us to retain our negative features? What if a genetic manipulation could, for example, eliminate envy?… I would not want to eliminate all of our bad features. I conjecture that that is partly because the negative traits are part of the package that makes human beings the particular valuable creatures that we personally cherish, and are therefore worth preserving as part of that package… (Cohen 2011, p. 209)^14^

On the basis of these reflections, Cohen suggests that the distinction he draws between particular value and personal value may be used to distinguish, respectively, between:

… the reason to preserve human beings (as they are)—that they are creatures that exhibit a certain form of value, and our (additional) reason to do so, which is that they are us. (Cohen 2011, p. 209)^15^

As the above quotes from Cohen intimate, he seems to believe that there are two separate reasons to refrain from carrying out human enhancements, reasons which correspond to the two types of value he distinguishes. First, he claims that we have a reason to preserve human beings as they are insofar as they instantiate a ‘certain form of value’. Cohen does not explicitly cash out what he means by this claim; however, what seems to be underlying the argument here is an appeal to something like the value of ‘human nature’, or of our shared human characteristics. On this interpretation, insofar as human beings can be said to share in a common nature, that nature possesses intrinsic value, or at least partly explains why human beings are valuable.^16^ Accordingly, the view that seems to be implicit in Cohen’s comments here is that even if our shared human nature could be improved in a manner which would increase the intrinsic value of human beings, we should refrain from doing so because human nature *as it is* also has particular value in abstraction from its intrinsic value (in the same way that Michelangelo’s David does).^17^ Call this *the argument from particular value*.^18^

Interestingly, if this interpretation of Cohen is correct, then it may offer bio-conservatives an attractive way of seeing human nature as having a distinctive value that does not face the standard problem of explaining why the relatively contingent and arbitrary features of the human species, selected by a blind evolutionary process, should be taken to have special value as many bio-conservatives seem to think. On the Cohen view, this contingency is not a problem. If we had been very different, it is that other nature that would have been valuable. Ours is valuable simply because it is the nature that *actually* exists, not because it is inherently special, or because there is some morally authoritative natural order that we are forbidden to trespass.

However, one problem with this interpretation of the argument is that what actually exists are *us,* that is to say particular extant humans who may be said to instantiate human nature, and not some abstract notion of ‘humanity’. This might be deemed problematic on one ground for the scope of the argument from particular value, since future humans do not actually exist to instantiate human nature (and thus particular value), and it is therefore not clear how the argument can extend to them. Unless we can make sense of ‘humanity’ as an existing entity, then this line of argument would only apply to changes we make to *ourselves*. However, the general point remains that, on this interpretation of Cohen, the conservative has grounds for objecting to the project of human enhancement, insofar as that project seeks only to enhance what is intrinsically valuable about human nature, whilst ignoring the particular value of human nature *as it now* *is*.^19^

The second objection to human enhancement suggested by Cohen is that to change our nature is to change something that possesses *personal* value; our additional reason not to improve human beings is that ‘they are us’. How can we make sense of the idea that humans beings are valuable ‘because they are us’? At the individual level, it seems relatively easy to cash this thought out; we clearly have a relationship to our own individual features that may be understood as a basis for each of us placing personal value on ourselves. However, it is not so simple to explain this claim at a collective level. One way of cashing out Cohen’s claim in this way would be to say that as well as sharing a common human nature, humans as a species have also developed that nature over the course of a shared biological history.^20^ It might then be argued that to seek to change ourselves by using enhancement technologies would be to fail to recognise the significance of our collective relationship to our own shared history, and the personal value that we, as a collective, place on it; on this interpretation of Cohen, we can collectively place personal value on ourselves as we are in view of this shared history that has made us the way we are^21^ Call this *the argument from personal value*.

As the above description intimates, there are two possible readings of the argument from personal value; the relevant reading of the argument will depend upon whether we understand the concept of personal value at the individual or collective level. In his brief remarks concerning enhancement, Cohen seems to have in mind primarily the enhancement of unborn children, since he refers only to the dangers of germline genetic manipulation (and not somatic interventions). However, consider first the argument as it pertains solely to the somatic enhancement of adult humans. Understood this way, the thrust of the argument from personal value seems to be that *individuals* may place personal value on their own features, so that even if it were possible to improve an individual’s nature through the use of, say, coercive pharmacological interventions, this would be to ignore the personal value that individuals may place on their own, flawed, natures.

This reading of the argument is somewhat narrow; understood this way, the objection relies on the thought that the individual herself places personal value on her own features as they are, and that an external agency is seeking to coercively impose an enhancement that would be insufficient to overrule this personal value. However, these are not the sorts of cases of enhancement that ‘liberals’ in the enhancement debate normally aim to defend. Such liberals primarily seek to defend against governments coercively *preventing* agents who have *chosen* to enhance themselves from doing so, when that choice arises from their recognising what they perceive to be a flawed or unsatisfactory feature which they desire to improve.

Nevertheless, this individualistic reading of the argument from personal value could still be used to object to governments coercively imposing certain enhancements—cases in which the enhancements were carried out on individuals despite the personal value that they place on their own features. However, this is clearly a limited sort of objection to enhancement technologies.^22^ Moreover, as well as being inapplicable to individuals who do not personally value certain aspects of their own natures,^23^ the above reading of the argument from personal value is also not applicable to the use of germ-line enhancements, since the unborn recipients of germ-line enhancements cannot personally value their own features in the way that this reading requires; after all, they do not yet exist in the sense that would be required for valuing their features.

However, Cohen seems to believe that what we have termed the ‘argument from personal value’ is also applicable to the use of germ-line enhancements. This suggests an alternative, broader reading of the argument pertaining to personal value at the collective level. On this broader reading, the thrust of the argument does not lie in the fact that the recipient of the enhancement herself personally values her own features; rather, we collectively place personal value on human nature insofar as it is the outcome of our shared biological history, warts and all. On this reading, the way humans actually are has a certain long history which is *our* history, and thus confers special personal value on how we biologically are. Those who seek to enhance themselves (or future people) could be accused of failing to see that they have this relation to their own nature.^24^ This seems to be part of what Cohen has in mind in his remarks on envy; his thought seems to be that we should place personal value on all aspects of our nature, the good and the bad, just because we are human and it is *our* nature under consideration.

To conclude this section, the intuitive force underlying the conservative objections to enhancement that Cohen suggests is the following: even if the technology to improve certain of our capacities becomes available, and even if proponents of enhancement are correct to claim that the changes that enhancements could bring about would lead to a better outcome, to carry out such enhancements would be to fail to appreciate the fact that human nature *as it is* not only bears a certain value, but is also valuable (in a particular and personal sense) by virtue of the fact that it is a value that exists and which we have a special sort of relation to (understood either at the individual or collective level). Accordingly, the conservative might argue that human enhancements should be avoided, whether they are coerced or freely chosen, in so far as they would serve to replace these special extant values.

## Critique

The previous section delineated the theoretical basis for the way in which the conservative might object to the use of human enhancement technologies. In view of this, it might seem, *prima facie* at least, that the conservative ought to oppose *all* forms of human enhancement technologies. However, closer inspection of the matter suggests that this is not the case. In the remainder of this paper, we shall show that if the objection to human enhancement based on Cohen’s conservatism is to have real plausibility, its scope must be limited in important ways.

### Overriding Values

By Cohen’s own lights, one limitation to the conservative objection arises from his caveat that the conservative bias is defeasible. Even if it may be important to preserve human nature as it is on the grounds of both personal and particular value, there may be other values which outweigh this bias. If so, then all that the objection shows is that we have reason to avoid enhancements whose benefits are not significant enough to outweigh these other values.

One candidate for a value which could overrule the particular and personal value of human nature, and one which is central to Cohen’s work elsewhere, is the value of social justice (Cohen 2000). Indeed, in his discussion of the conservative bias, Cohen himself states that justice always trumps it (Cohen 2011, p. 224). To illustrate, if it were the case that social justice could be more effectively achieved if certain members of society were enhanced (perhaps so that they had equal access to opportunities afforded to those with higher capacities), then it might again be a requirement to enhance them in the interest of justice. Indeed, in view of the fact that Cohen defended luck egalitarianism, it may be possible to go even further than this. In a paper on egalitarian justice, Cohen states that “… the primary egalitarian impulse is to extinguish the influence on distribution of both exploitation and brute luck” (Cohen 1989, p. 908). Genetic injustice seems a paradigm instance of just that; clearly, having less innate talents than others is obviously outside of our control. So it seems to follows that luck egalitarianism should support very strong forms of genetic enhancement which seek to mitigate these genetic injustices.^25^ Coupled with his claim that injustice always trumps the conservative bias, it seems that we get a very powerful argument *for* certain forms of genetic enhancement.^26^

A further candidate for a value which might outweigh the conservative bias is the value of preserving human existence itself. Savulescu and Persson have argued that we have a moral imperative to morally enhance members of society in order to reduce the ever increasing probability of the human race being destroyed by a small group of individuals (Savulescu and Persson 2012). Accordingly, if the only way in which we can preserve the human species is by changing human nature in order to enhance certain characteristics which will make the preservation of the species more likely, it seems incumbent upon us to do so, even if it means overriding the personal and particular value possessed by our flawed natures.^27^

It might be argued that rather than overriding the conservative bias, the above case of preserving human existence is an example where we have more reason to conserve one extant value (i.e., the survival of the human species) than we do to preserve another extant value which is mutually exclusive (i.e. the value of our current characteristics). This thought leads us to another possible limitation of Cohen’s objection to enhancement technologies; namely, it does not seem to be effective against those enhancement technologies which serve to conserve extant valuable aspects of human characteristics. It is to this issue that we now turn.

### Enhancements that Conserve

The second thing to acknowledge is that, again by the conservative’s own lights, not all enhancements will necessarily be susceptible to the conservative objections delineated above. The main thrust underlying both of these objections is Cohen’s thesis that we have a reason to conserve what has value, whether it is particular or personal value at stake. Accordingly, if an enhancement can be regarded as merely conserving what is valuable about human beings, it seems that Cohen’s arguments could in fact be used to *support* the use of certain enhancements.

To use one of Cohen’s own examples (which is not related to his comments on enhancement), Cohen refers to the value of All Souls College in Oxford as an institution with a particular identity, and claims that the college should not be changed so as to bring about more value (say by being changed to make it capable of promoting research better) if bringing about such a change destroys the very identity of the college. The notion of identity at work here is not the purely metaphysical sense of identity that is often at work in questions of personal identity in the philosophical literature. Rather, Cohen points out that he uses the notion of identity in a “… vaguer but very important socio-cultural sense” (Cohen 2011, endnote 6).^28^

For our purposes, the crucial part of Cohen’s discussion of this example is his claim that his concern in this case is that “All Souls remains what (but not therefore exactly *as*) it is” (Cohen 2012, p. 169); Cohen here is conceding that it is possible to change an entity without changing what it is in the manner that would threaten its socio-cultural identity. In view of this, it seems plausible to suggest that Cohen would surely embrace restorations to All Souls whose purpose is to preserve the College, presuming that these changes do not threaten its unique and valuable identity; such changes may be said to change the college from being *as* it is, without changing *what* it is.

We can apply a similar thought to the enhancement of certain features which do not seem to threaten to alter what seems valuable about human nature. As we stated above, Cohen himself is not explicit about what it is that is supposed to be valuable about human beings. However, even without a detailed discussion, it seems plausible to claim that at least one sort of enhancement that may be viewed as *conserving* what is valuable about human beings, rather than replacing something which has particular or personal valuable—namely, life-extension technologies.

Consider the following example:

Edward’s father has just died, at the age of 72. Although he had enjoyed his life, in his final years Edward’s father often told him how he regretted the many things that he had not done. This prompts Edward to take a course of drugs that will extend his life by 30 years. Moreover, these drugs will also stop the aging process, ensuring that Edward’s cognitive and physical traits will not diminish over the extra time that he will now live.

If human life has value, then surely extending Edward’s life in this way would *conserve* existing value. Moreover, normal ageing brings about the loss of many valuable and valued human capacities, such as the loss of cognitive, sensory, emotional and motor capacities; the preservation of these would require human enhancement. Protecting against normal cognitive decline, normal loss of hearing and sexual potency, normal physical infirmity requires that we enhance human capacities just as we would have to enhance and replace stone work that ages on a building.

Similarly, we might also acknowledge that it is a painful, familiar truth that love withers and dies. Nearly 50 % of marriages end in divorce and there may biological as well as sociological reasons for this (Savulescu and Sandberg 2008; Earp et al. 2012). Such relationship break-up can have bad effects for all concerned. The prospect of biologically maintaining attachment and enhancing pair bonding for promoting long term relationship stability might be welcomed by some. Coupled with “environmental enrichment” and a vigorous “love exercise programme”, it is hard to see a conservative objection to such love drugs. Indeed, enhancing the stability of marriage is one of the central goals of many conservatives.

If this is right, then life-extension and love-enhancing technologies may again be regarded not as replacing something that is valuable about human beings; rather, this sort of enhancement may be regarded as conserving something that is valuable about human life, but which we often lose in natural circumstances.

One possible objection to this alleged limitation to the scope of Cohen’s argument, and one that would not apply to the analogous case of preserving All Souls College, would be if there was something inherently valuable about the current human life-span (Kass 2003), or the current life span of loving relationships. But this objection is implausible; why should we think that there is anything special about the proverbial ‘threescore and ten’ average life expectancy in the developed world? After all, a life expectancy of this length is a very recent development in human history; life expectancy has dramatically increased even over the last century due, in part, to unprecedented developments in medical technologies.^29^ Even without the use of enhancement technologies, we can expect life expectancy to increase on the basis that medical technology and science is likely to improve.^30^ Through most of human history, most people lived substantially less than 40 years. And we doubt bioconservatives would wish to maintain the current life span of relationships, which has a median of 7–10 years (Savulescu and Sandberg 2008).

It thus seems that there are other cases in which an enhancement could be used to conserve something of value. Consider the following example, closer to Cohen’s heart:

Charles has been married to Charlotte for a number of years, but their relationship is under strain. Although Charles and Charlotte both love each other, Charlotte finds it difficult to deal with Charles’ pathological envy. Charles loves Charlotte and wants to change his ways, but neither anger management classes nor psychotherapy have reduced his envious tendencies. Charles therefore decides to take certain drugs which have been shown to reduce envy.

In this example, although it is possible to regard Charles’ attempts to rid himself of envy as an attempt to destroy an existing feature that has personal value, it seems plausible to claim that Charles’s motive in undergoing the enhancements is that he wants to conserve the particular and personal value instantiated by his marriage to Charlotte; it seems that this latter value is far greater for Charles than the (supposed) personal value of his envy.

In this case, it is also noteworthy that the couple are not engaged in a general project of removing envy from humanity but in reducing it in a particular case, in order to preserve something of particular value. Since it would be good for them to do this using non-biomedical means, for example in couples’ therapy, it is hard to see how it could be wrong to use biomedical means if these are more effective, or these supplement environmental manipulations.

As we have suggested in these two sub-sections, the conservative bias cannot ground a sweeping objection to *all* forms of human enhancement, even if it still serves to highlight the fact that changes to our existing nature, while sometimes justified, should still be a source of some regret, a point that proponents of enhancement often overlook. It might still be thought, however, that enhancements that conserve existing value are a special case, and that there will be enough cases where the conservative bias will not be clearly overridden by other values. We turn next to examples of that sort.

### Organic Change

As we pointed out in considering Cohen’s discussion of the All Souls example, Cohen’s conservative concern is to preserve *what* exists, but not necessarily *as* it is. What this seems to imply is that the conservative should find certain changes permissible so long as those changes are *respectful* to the value that currently exists.

To return to the example of All Souls, the college has undeniably changed in important ways over the centuries; for instance, new buildings have been built, and plumbing and electricity have been installed. These changes have surely made the college better in numerous ways. Crucially though, these changes do not amount to changing *what* All Souls is with regards to the sense of identity that Cohen has in mind. It seems plausible to claim that part of the reason why they have not is that these changes were brought about in a manner that respects existing value. In contrast, we can easily think of other changes to All Souls that would be genuinely compromising; for instance, suppose that a beautiful but incongruous modern glass tower was built in the middle of the main quad. The conservative would understandably lament this rude intervention, but that does not mean that he must lament *all* changes to the college that go beyond those essential for its strict preservation; there is no good reason for him to lament those changes that improve things while still respecting existing value.

In a similar vein, something that may strike us when we consider how Cohen’s defence of the conservative bias relates to the enhancement debate is the fact that humans have improved themselves in several ways throughout history, and in ways which do not cause us concern. One clear example of a way in which humans have changed over the course of their history is the improvement in their cognitive capacities. Clearly, we are far more cognitively advanced than our early ancestors. Yet one need not even go this far back to witness our cognitive improvement; according to the well documented Flynn effect, average IQ scores have been steadily increasing over the past few generations (Flynn 1987). In a similar vein, Steven Pinker has documented how humanity’s tendency towards violent behaviour has gradually, but significantly reduced over the course of our history (Pinker 2012). Few, if any, would deny that both of these changes have been for the better. Presumably, Cohen would not mean his conservatism to extend to these changes in human nature^31^; there is nothing wrong in these changes, nor should we lament them. It seems to us that these changes seem less threatening because, like the changes that All Souls has undergone over the years, they do not seem to disrespect existing value.

This raises the question of *why* these sorts of change are compatible with respecting existing value. One salient difference between the natural changes listed above and the sorts of enhancements that Cohen seems to find worrying is the *length of time* over which the changes would take place. Consider first the case of cognitive enhancement. Rather than human IQ gradually increasing over generations, the prospect of enhancement promises an almost immediate and radical change to a particular individual. Similarly, whilst our violent tendencies may be decreasing over generations, this seems to be different from someone immediately eradicating violence, envy, or a gloomy outlook by taking a pill.^32^

However, the fact that a change is merely gradual is neither necessary nor sufficient for its being respectful to existing value. To return to the All Souls example, if one built the beautiful but incongruous modern glass tower in All Souls, but built it very slowly, one would still fail to respect the value of All Souls’ identity. On the other hand, installing electricity in a way that is unobtrusive seems compatible with respecting All Souls’ identity, even if it is done in a week.

As such, rather than claiming that a change respects existing value if it is gradual, we shall use the term ‘organic’ to denote those changes that seem to be compatible with respecting existing value. It is not easy to spell out precisely the exact contours of what makes a change organic in the sense that we are employing here. However, we can begin to elucidate this somewhat opaque concept by recalling the importance of history to personal value. We argued above that the thrust underlying our placing personal value on some entity is our shared history with that thing; as such, we may say that a change to some entity will fail to be organic if it somehow ruptures our sense of heritage with that entity.

In many cases, it is fairly easy to classify some change to a thing as organic or not. All Souls’ identity is not threatened by the move from quills to typewriters and from typewriters to computers. But it will be threatened if, say, the chapel was turned into a gym. Similarly, adding a new quad to the college would be a significant change, but it might still be organic if the quad preserved the existing architectural style of the college; it will not be if its look is entirely modern. The operative idea seems to be that of continuity in form and function—this is obviously a matter of degree, and there will be plenty of borderline cases. The weight given to different aspects of an entity’s current form and function depends on their role in its ‘social-cultural’ identity, and that, we suggest, depends at least in part on its shared history.^33^

Although the reflections on the All Souls case suggest that a change’s being gradual is neither necessary nor always sufficient for its being organic, it might be the case that the immediate nature of some changes will be sufficient for its being *inorganic* and thereby disrespectful of existing value. There is an interesting issue here of when change needs to be gradual to respect existing value. Perhaps some significant or radical changes (bearing on core features or form) cannot be done in an organic way if done quickly. We shall not pursue this point here. However, the discussion of this section suggests that the conservative objection is most easily aimed at those enhancements which *radically* alter some aspect of our natures in a manner which we may intuitively describe as ‘inorganic’, in the sense that it ruptures our sense of heritage with the extant object. Clearly then, one aspect of a change that can sometimes serve to render it inorganic is if it takes place over a very short period of time. As such, we may say that Cohen’s conservatism give us a reason, to be wary of immediate, radical enhancement, since the immediacy of certain radical changes can render them ‘inorganic’ and thereby disrespectful of existing value.

At this point though, we might recall that Cohen’s thoughts concerning his plastic man case suggest that even *gradual* enhancements ought to be avoided if they are sufficiently radical. In the next section, we shall consider what might constitute a non-radical enhancement.

### Non-Radical Enhancements—Changes that Preserve Essential Features

As we have already seen in Cohen’s discussion of the All Souls example, it seems that an integral part to a change’s being organic (and thereby compatible with respecting extant value) is that the change does not alter those essential features of the entity which undergird its socio-cultural identity. Just as Cohen pointed out that All Souls has an identity which ought to be preserved in view of its particular and personal value, one might claim that we would not be the individual persons we are if it had not been for what we take to be our defects; they, as well as what we take to be our strengths are essential features that constitute *who* we are. The argument, then, might be that we should not seek to change ourselves too much for the better for fear that we may in fact change who we actually are.

This thought bears some similarity to Robert Adams’ arguments concerning the value we place on our own lives. Adams argues that we have reason to prefer our own actual life, if that life is good, rather than lives which could have been better. That is, we should prefer our actual life if those different lives would have been “too thoroughly different” (Adams 1979, p. 60) in terms of the projects, friendships and attachments that characterise them, and that we now actually care about. And given that what matters to us now are certain projects, friendships and goals, we should not regret the fact that we have our actual lives, and not better but different lives in which these things did not matter, just as we can be happy that we exist, and not that other happier individuals instead of us. For example, Adams argues that one can have reason to choose freedom over happiness or some other objective value, without claiming that the free society is better: “It is enough that the type of society for which one strives is good, and worth loving” (Adams 1979, p. 63).

It might thus be claimed that radical changes that undermine (or otherwise compromise) our socio-cultural identity fail to respect the existing value of our current human nature. However, the objection as it has this far been stated is not convincing. Once again, it seems clear that individuals often seek to improve themselves during their lives without threatening their sense of identity. Indeed, as we pointed out in our discussion of the All Souls example, we often improve things in order to *conserve* their identity; presumably improvements do occur in All Souls College that do not undermine the particular and personal value of its socio-cultural identity.

Is there any way in which enhancement technologies might be disanalogous? Consider first the case of life extension enhancements. Here it seems that the enhancement would serve to preserve the recipient’s identity in the sense that Cohen is concerned with rather than to threaten it; after all, one does not have an identity in the sense that Cohen alludes to after one has ceased to exist.^34^ Moreover, it seems plausible to claim that Edward’s socio-cultural identity is at least partly associated with certain psychological components such as his memories, and his beliefs. It seems plausible to claim that life extension could serve to conserve these identity constituting psychological components, which often fade in the later years of life. Most strikingly, in some cases of intervening in dying or fading love, enhancement may seek to promote what both parties cherished, their shared history, against factors which may be partly outside of their full control.

Having stated this, it seems that other enhancements do not merely *conserve* certain capacities which are linked to the agent’s identity, but seek to improve upon them. Might this be more problematic? It is difficult to see why. After all, we often seek to improve ourselves through natural means such as education without fear of threatening our socio-cultural identity.

It might be argued that the conservative objection against enhancements has more force on the collective understanding of the argument from personal value, where enhancements are understood as threatening some ‘species identity’ or human nature that needs to be preserved. We believe that this is a more problematic proposal, given the difficulties associated with these notions (Nielsen 2011), and it is not entirely clear what Cohen has in mind when he writes about the “package [of features] that makes human beings the particular valuable creatures that we personally cherish”. However, that is not to say that this objection is completely lacking in content. It seems plausible that some enhancements might pose a threat to certain agreed central features of humankind. For instance, Robert Sparrow has suggested that we could alter the human race to ensure that all new-borns were female, and that this could qualify as an enhancement (Sparrow 2010).^35^ It seems that we could plausibly regard such an ‘enhancement’ as undesirable not because such a change would be ‘against nature’, but because it would represent a fundamental and radical disjunct between the human species (or even the ‘human form of life’) as it existed prior to the intervention and as it would exist following it. The conservative bias can give us reasons to reject such radical transformations of human nature even if (as Sparrow assumes) they promise to lead to a better outcome. It gives us, one might also say, reasons to be *partial* to aspects of human nature as it currently is—but only within limits.^36^

Another aspect of an enhancement that the conservative might claim is morally relevant is the manner of the enhancement. For example, Cohen’s repulsion in the face of the plastic man example suggests that he regards the change here as inorganic despite the fact that it is gradual. There is some intuitive pull to this idea; it seems that physically replacing naturally developed parts of one’s body with synthetic substitutes represents an inorganic change, not only literally, but also in the sense that we employ here. Whilst it would be interesting to explore the theoretical basis for why such a change seems to be inorganic, space does not allow for a full investigation of this matter here. Needless to say, however, this is a rather extreme form of enhancement. By contrast, although, say, mood enhancing pills might not be ‘natural’, it seems more plausible to claim that the change they bring about is more compatible with respecting existing value, since such pills serve only to modulate existing neural substances. Perhaps what this suggests is that if we accept the conservative bias then we have some reason to prefer enhancements which do not involve synthetic substitutes, even if they would lead to a greater benefit. This strikes us as the most interesting implication that Cohen’s conservatism has for the enhancement debate, but its significance will depend on whether the intuitive but opaque notion of ‘organic change’ can be fleshed out in a persuasive manner—a question that lies beyond the scope of this paper.

### The Problem of Disloyalty

A related worry which can be read into Cohen’s thought here, and which Bernard Williams explicitly pressed, is the worry that enhancements might endanger what Williams calls our “*ethical* identity as a species” (Williams 2008, p. 152).^37^ The idea here is that in seeking to enhance ourselves, we are seeking to distance ourselves from humanity as it now exists, expressing a self-hatred, or hatred of humanity in its current state.^38^ Similarly, Cohen might claim that seeking to enhance humanity represents a disloyalty to the particular and personal value that human kind currently instantiates.

Sometimes, seeking to improve upon something of value does express a form of disloyalty. Cohen makes this point by discussing the lyrics of two love songs; he points out that the sort of loving attitude that we seek is one in which I am “… loved not as a good bearer of my virtues, but as *the* bearer of them that I am” (Cohen 2012, p. 154). This seems right in part. When we truly love someone, that person becomes irreplaceable to us; finding someone else who is more beautiful, charming, and intelligent than them should not lead us to abandon our beloved in their favour. Such a person would be, as Adams puts it, “too thoroughly different.” Indeed, it seems plausible to claim that one of the reasons that adultery is so painful for the betrayed party is that the adulterous act suggests that one is replaceable; the adulterous act indicates that one was only ever loved in virtue of bearing certain values (which others may bear more of), rather than by virtue of the unique person that one is.

However, although the fact that something is irreplaceable might entail that we cannot improve our lot by replacing it, this does not entail that the irreplaceable entity cannot *itself* be improved, or that seeking to improve it must express a detrimental attitude towards that object. For instance, consider the practice of child rearing. As Sandel points out, following May, parental love involves loving and accepting the child unconditionally as they are, whilst also seeking to transform and improve the child for the sake of the child’s well-being (Sandel 2007, pp. 49–50).^39^

It seems, then, that there is an important difference between replacing something of value in order to improve one’s lot, and improving something of value that one already has—not to mention improving it *for its own sake*. Indeed, it seems that whilst the former often does seem to express a form of disloyalty, the latter can even be an expression of loyalty to something of value. Compare the case of adultery with the case of Charles and Charlotte. The case of adultery clearly involves disloyalty; the adulterer simply replaces the value her spouse bears with value borne by another. However, in seeking to change Charles by reducing his envious tendencies, or in consensual love enhancements in general, it is not at all clear that Charlotte is being disloyal to the particular and personal value that Charles instantiates. Indeed, if her motive for doing so is to safeguard her marriage to Charles, then this is surely an expression of her loyalty to, and love of Charles. A loving attitude is surely compatible with a hope for change, even if it is incompatible with a desire to replace. Although seeking to replace a valuable existent might be contrary to the conservative bias, it is not at all clear that improving the value of the same existent must be.^40^

More generally, in seeking to change certain aspects of human nature through the use of enhancement technologies, it seems more accurate to say that we are seeking to improve human nature rather than to simply replace it wholesale; and it is not clear why merely seeking to improve ourselves must indicate a hatred of what we now are, or something “ghastly”. Indeed, it seems plausible to claim that part of what is so valuable about human nature is our capacity and readiness to improve ourselves. The enhancement project then may be regarded not as an expression of self-hatred, but rather of self-realisation.

## Conclusion

In this paper, we have argued that Cohen’s views on value suggest a new line of argument against enhancement that is in several ways clearer and superior to existing objections. However, we have argued that this argument fails to offer a basis for a strong sweeping objection to enhancement. First, Cohen’s conservatism can positively support some forms of enhancement that aim to preserve existing/personal value. Cohen’s arguments about particular and personal value serve to highlight the importance of an extant valuable entity’s history to its current value; however, we can often preserve history while embracing enhancement. And enhancement can be a part of *creating* history. Importantly, Cohen’s view might have quite radical implications for debates relating to future generations. It appears to support giving priority to enhancement and extension of existing lives, rather than bringing into existence new generations. And it appears to prioritise the interests of existing individuals over possible future individuals.

Moreover, it is not clear that Cohen’s arguments are sufficient to render those enhancements which do not simply preserve existing values morally impermissible. Rather, they suggest some possible constraints on the modality of legitimate (or desirable) enhancements. As we highlighted above, Cohen himself claims that the conservative bias is defeasible; we have suggested that the values of social justice and the value of preserving human existence can override the conservative bias. Finally, we have suggested that the conservative need not object to those enhancements which bring about change in a manner which respects values that currently exist; as such they have no reason to resist enhancements which are organic, and do not alter the essential features which undergird our particular human identity.

## Footnotes

1 This aspect of utilitarianism has been famously criticized by Rawls (1974), Williams (1973) and Stocker (1976).

2 Notice that while the bride’s relation to that dress may be thought to literally confer (extrinsic) *value* on that dress—what Cohen calls ‘personal value’—it is less plausible to think of ‘particular value’ as some *extra* value that something of intrinsic value possesses simply in virtue of actually existing. Cohen is better understood as making a claim about *how* it is appropriate to *value* existing things that bear intrinsic value, not about some *further* kind of value. However, to simplify presentation, we will follow Cohen in writing about ‘particular value’.

3 Understood in this way, the reasons generated by personal value are primarily agent-relative. This means that some object may possess opposing personal value to different people. For example, another woman who was in love with the bride’s husband may personally *disvalue* the wedding dress. In such cases, it may not be possible to determine the appropriate course of action only by appealing to the concept of personal value. Other considerations, such as the considerations relating to right we mention below, will be needed to resolve such conflicts. We are grateful here to an anonymous reviewer for *The Journal of Ethics* for pressing this point.

4 For a classic account of this sort of perspective, see Nozick (1974).

5 We are grateful to an anonymous reviewer for *The Journal of Ethics * for suggesting this alternative approach. We note, however, that such an approach may not explain the *bride’s* reasons for treating that dress differently from other things she owns—on such a view, her partiality to that dress is merely a subjective relational state. And there are other relevant forms of apparent extrinsic value that will not be easily accommodated by this approach, such as Shelly Kagan’s claim that the pen that Lincoln used to sign the Emancipation Proclamation is of value in itself, because of its distinctive historical role (Kagan 1998).

6 Cohen (1995). Notice, moreover, that some prominent Kantians have defended broadly related claims about extrinsic value. See Korsgaard (1983).

7 This conservative bias is of course relative to a point in time. If Michelangelo did destroy David, and created Eve, then *Eve* would now possess particular value, and it could be similarly wrong for Michelangelo to destroy Eve in order to create an even better third statue.

8 As Cohen also points out, his argument does not entail that the conservative ought to be biased against *all* new things; after all, it is possible that new values can be brought into existence without destroying the old, and it is the latter which the conservative bias laments. (Cohen 2011, p. 213).

9 Notice that Cohen’s point here is not merely that the dress has *extrinsic* rather than intrinsic value; it also lies in its value as a *particular* entity—it would be awry to destroy the wedding dress if it could be replaced with another thing of similar sentimental value.

10 The point that Cohen is making here seems to be an extension of the influential view that in loving relationships it is the particular person herself who is loved and not the person’s characteristics per se. See Nozick (1974, pp. 167–168) for an early formulation of this point. We consider Cohen’s own discussion of love, below.

11 When Cohen writes about ‘valuing the valued’, this suggests that certain things possess (personal) value because we *value* them. It is implausible, however, that Cohen means that *anything* that anyone values thereby acquires personal value. As his examples suggest, some objective relationship to the object is needed to ground that valuing—perhaps a shared history, as we suggest. Might such an objective relationship confer personal value on something *even if one does not value it*? This is less clear. We shall largely assume that both (objective) relationship and (subjective) valuing is required for personal value, but we shall also consider cases where the two come apart.

12 Interpreted in this way, Cohen’s claims about personal value can again be seen as an extension of the more familiar view that our relationship with family and friends justify our partiality towards them, where such relationships are at least partly constituted by a shared history. See e.g. Kolodny (2003). This would explain what would be wrong about replacing the dog Jessie with the puppy Sally.

13 One particular worry here is that the conservative bias seems suspiciously similar to status quo bias. Cohen does briefly attempt to address this worry (on p. 225), but his arguments here are not wholly persuasive. It seems that this worry applies with greater strength to ‘particular’ value than to ‘personal’ value.

14 As an anonymous reviewer for *The Journal of Ethics* pointed out, we need not rely on the conservative bias in order to object to an attempt to eliminate envy. We could also do so on the basis that envy is closely related to other desirable conative states such as competiveness, and it might be impossible to eliminate one without eliminating the other.

15 This claim again echoes the late Bernard Williams’ attempts to justify speciesism and the human prejudice over according equal value and consideration to other life forms and species. As Williams writes, “Told that there are human beings trapped in a burning building, on the strength of that fact alone we mobilise as many resources as we can to rescue them” (Williams 2008, p.142).

16 On another interpretation, we humans possess intrinsic value because of more generic features, such as self-consciousness, rationality and moral capacities. Other aspects of human nature (such as being made of flesh) are endowed with particular value not because they themselves possess intrinsic value, but simply because they are the contingent features of the particular beings that we happen to be. We could still be rational even if we completely transformed our nature, but in doing so we would be failing to respect this particular value.

17 Whilst an entity’s having particular value depends on its bearing intrinsic value, the entity’s particular value is to be conceived of in abstraction from the intrinsic value that it bears.

18 This would also appear to ground Williams’ ‘human prejudice’. See Williams (2008).

19 Of course, a person would still exist following an enhancement, and their enhanced nature would presumably have some particular value on the basis that it now exists. This is compatible with Cohen’s view. Whilst such an enhancement would involve the creation of something with new particular value, it would necessarily involve the destruction of that which previously existed and instantiated particular value. Reasons generated by particular value are relative to time. At any given point in time, we have reason not to replace something of particular value with some new entity of greater intrinsic value. But if we ignore this reason, then the replacement would itself come to possess particular value, and we would now have reason not to replace *it* with something *even* better. There is no inconsistency here.

20 In questioning the existence of any cosmic point of view, Williams remarks, “There is certainly one point of view from which they are important, namely ours: unsurprisingly so, since the “we” in question, the “we” who raise this question and discuss with others who we hope will listen and reply, are indeed human beings. It is just as unsurprising that this “we” often shows up within the content of our values. Whether a creature is a human being or not makes a large difference, a lot of the time, to the ways in which we treat that creature or at least think that we should treat it.”

21 We are aware that there are some problems with this interpretation of Cohen’s claims here; first and foremost, it is not clear that all aspects of our shared history have value, because our shared history has also led us to develop undesirable traits. However, what is crucial for the point that we are making here is that our current nature is valuable, and we can describe ourselves as placing *personal* (as well as particular) value on it by virtue of the fact that it developed, warts and all, over the process of a shared history.

22 However, as an anonymous reviewer for *The Journal of Ethics* pointed out, it might be the case that initial uses of enhancement technologies might be coercive in institutional and political domains (such as the military). As such, whilst the argument above is limited in scope, it is nonetheless important. Thanks to the anonymous reviewer for making this point.

23 As we noted in note 11, Cohen’s claims about personal value can also be understood in a more objective manner. On this reading, our relationship to our particular nature endows that nature with personal value for us *even* if we do not in fact place value on it. Understood in this more demanding way, personal value could generate reasons against enhancement even for individuals who are keen to radically transform their own nature.

24 There are some interesting differences here between collectively placing personal value and an individual placing personal value on something. Whilst it is unclear how an agent could fail to see their own specific relation to some object without undermining the thought that the relationship itself is of importance (though see note 23), it is easy to see how a minority of a collective could fail to see the importance of the collective’s relationship to a shared history without undermining the actual importance of the shared history to the collective as a whole.

25 Dworkin (2003) also seems to essentially endorse this conclusion.

26 Of course this would be compatible only with enhancement that is enjoyed by everyone; it would strongly count against letting rich people enhancing their children if poorer people cannot.

27 This raises the interesting point of whether the conservative bias could also be overruled in order to prevent non-human animals and/or existing human cultures from becoming extinct. Presuming that we valued the existence of these cultures and species, it seems that there would be a good case in favour of this. We are grateful here to an anonymous reviewer for *The Journal of Ethics*.

28 When referring Cohen’s conception of identity to human agents, the sense of identity he uses seems to have more in common with DeGrazia’s conception of narrative identity than that of personal identity. See DeGrazia (2005).

29 See UK house of Commons research paper (1999) on this matter.

30 A conservative of Cohen’s ilk might respond to this point by claiming that the Cohen’s view protects value as it actually exists *now*. Accordingly, it might be claimed that the conservative does not need to show there is anything special about our current lifespan; it’s enough that it is what exists. However, this reply has limited force. After all, it follows from it that there was something slightly regrettable in moving from our past shorter lifespan to the current one. Yet this badness seems to have no moral weight against the measures that were taken to previously increase our lifespan. *Mutatis mutandis*, this form of value cannot weigh against extending lifespan even further.

31 It might be claimed that Cohen would want to extend his conservatism in this way, but allow that the benefits are large enough to outweigh our reason to conserve our e.g. aggression. If so, then the reply to this is again that this should also apply to further enhancements.

32 It should be acknowledged that Cohen explicitly denies that his view implies that the longer some valuable thing exists, the more ‘particular’ value it has. However, this is compatible with the point we are making here about gradual change. The point we are making is that gradually changing something is compatible with respecting the value that currently exists; this does not imply that the longer a particular entity exists the more particular value it has.

33 Michael Stocker also appeals to organic wholes. He uses several examples of’ ‘mixes of different goods’ to show that the value of any good is dependent on determining whether the ‘organic whole’ to which it belongs is better than another whole to which same good might belong. His conclusion is that we do not sum the good to find the better, but often must evaluate the better before we can determine the good. In this sense, maximization is parasitic on other views of the ‘better’ for its definition of the ‘good to be maximized’ (Stocker 1990, p. 295).

34 This discussion does not, of course, have any bearing on the numerical sense of identity which is not Cohen’s concern.

35 Sparrow takes this to be a reductio of the enhancement project.

36 Many authors have argued that we can be justified in being partial to our family and friends, and give them some priority over complete strangers (see e.g. Cottingham 1986). What we are suggesting now is that, seen in this way, this application of Cohen’s conservatism amounts to an extension of this partiality to human nature itself. But again such ‘species partiality’ will be outweighed when the benefits are very significant, on when it is trumped by weightier moral considerations. In Williams’ terms, the justification for the human prejudice has limits.

37 Emphasis added.

38 A related argument is found in the disability debate. See McMahan (2005) for discussion.

39 Interestingly, it might be claimed that good parenting might require parents to rid themselves of desires to ‘hyperparent’ and mould the child to one’s expectations. This would surely be an enhancement acceptable to bio-conservatives.

40 We concede, however, that there must be a limit to such a desire to change, since loving someone as a particular, yet at the same desiring ardently that they be *completely* different, seems awry.
